# Biochemical and Molecular Characteristics of a Novel Hyaluronic Acid Lyase from *Citrobacter freundii*

**DOI:** 10.3390/foods11131989

**Published:** 2022-07-05

**Authors:** Xinyue Li, Fang Li, Junhao Ma, Mingjun Li, Xi Lei, Xianghua Tang, Qian Wu, Zunxi Huang, Rui Zhang

**Affiliations:** 1 College of Life Sciences, Yunnan Normal University, Kunming 650500, China; 2023120024@user.ynnu.edu.cn (X.L.); 2123120025@user.ynnu.edu.cn (F.L.); 2123120052@user.ynnu.edu.cn (J.M.); 2123120047@user.ynnu.edu.cn (M.L.); leiandxi@163.com (X.L.); tangxianghua@ynnu.edu.cn (X.T.); wuqian@ynnu.edu.cn (Q.W.); huangzunxi@ynnu.edu.cn (Z.H.); 2 Engineering Research Center of Sustainable Development and Utilization of Biomass Energy, Ministry of Education, Yunnan Normal University, Kunming 650500, China; 3 Key Laboratory of Yunnan for Biomass Energy and Biotechnology of Environment, Yunnan Normal University, Kunming 650500, China; 4 Key Laboratory of Yunnan Provincial Education, Department for Plateau Characteristic Food Enzymes, Yunnan Normal University, Kunming 650500, China

**Keywords:** polysaccharide lyase family 8, low molecular weight unsaturated oligosaccharides, antioxidants, hyaluronic acid

## Abstract

The Gram-negative strain of *Citrobacter freundii,* YNLX, has the ability to degrade hyaluronic acid. In this study, we expressed a *C. freundii* hyaluronic acid lyase, from polysaccharide lyase family 8, in *Escherichia coli*. The purified recombinant enzyme (rHynACF8) showed a substantially higher cleavage activity of hyaluronic acid than chondroitin sulfate. We found that its optimal pH and temperature are 5.5 and 35 °C, respectively. In addition, the enzyme activity was not notably affected by most metal ions. *K_m_* and *k_cat_* of rHynACF8 towards HA were 1.5 ± 0.01 mg/mL and 30.9 ± 0.5 /s, respectively. rHynACF8 is an endo-acting enzyme. Its cleavage products had dramatically increased antioxidant activity than hyaluronic acid in vitro (*p* < 0.001). As the molecular weight of hyaluronic acid decreased, the intramolecular interactions among antioxidant functional groups were removed; in the process of the cracking reaction, new double bonds formed and conjugated with the carbonyl group. We presumed that the structural change is the critical factor influencing antioxidant capacity. Overall, we found that rHynACF8 from Gram-negative bacteria with metal ion resistance, indicated the relationship between the function and structure of its antioxidant cleavage product.

## 1. Introduction

Hyaluronic acid (HA) is a glycosaminoglycan that naturally occurs in living organisms. It is widely used in medicine, health care, and food [1]. The structure, functions, and biological activities of HA are closely related to its molecular weight [1,2,3]. High-molecular-weight HA is used for moisture retention, lubrication, and osmotic adjustment; thus, it is used as a dietary supplement to repair cartilage degeneration. However, low-molecular-weight hyaluronic acid is more easily absorbed by the human body; it is involved in many physiological functions, including accelerating wound healing by scavenging free radicals, promoting epithelialization and neovascularization, and remodeling collagen [4,5]. The enzymatic degradation of hyaluronic acid may be an optimal method for preparing bioactive low-molecular-weight hyaluronic acid oligosaccharides, due to the high efficiency and environmental friendliness of the process [1].

Hyaluronidases are a class of glycosidases that can degrade hyaluronic acid and part of glycosaminoglycan into oligosaccharides [6]. Initially, this class of glycosidases was regarded as a “diffusion factor” in animal extracts, promoting increased diffusion of subcutaneous vaccines, dyes, toxins, etc. These glycosidases were subsequently identified as hyaluronidases [7]. Based on the specificity of degradation products, hyaluronidases are classified as hyaluronic aminoglycosidases (EC 3.2.1.35), hyaluronic glucuronidases (EC 3.2.1.36), and hyaluronic lyases (EC 4.2.2.1) [8]. In the CAZy database (http://www.cazy.org/, accessed on 1 June 2022), hyaluronidases are divided into different glycoside hydrolase (GH) or polysaccharide lyase (PL) families. The enzymes in a family have similar sequence identities and structurally-related catalytic activities [9]. Combined with these two classification methods, hyaluronidases from microbial sources belong to PL8 and PL16 hyaluronic lyases, except for two PL56 hyaluronic aminoglycosides that are sourced from *Penicillium* spp. [10,11].

Hyaluronic acid lyases cleave HA to produce unsaturated double bonds, which had absorption peaks under ultraviolet in an eliminative mechanism. To date, all hyaluronic acid lyases that have been experimentally characterized as PL8 have been isolated from microbiology, but they have relatively large differences in their catalytic efficiency. Most of the enzymes are from Gram-positive genera, including *Streptococcus*, *Staphylococcus*, *Streptomyces*, *Microbacterium*, *Bacillus*, and *Thermasporomyces* [12,13,14,15,16,17,18]. Only HCLase (i.e., HA and chondroitin sulfate lyase) is from a Gram-negative *Vibrio* sp. FC509. HCLase from *Vibrio* sp. FC509 has the highest specific activity toward HA, but is sensitive to metal ions [19]. Hyaluronic acid lyases from Gram-negative bacteria have rarely been explored at the functional level, and the mining of enzymes from Gram-negative bacteria may identify good-quality resources for basic research and potential applications.

In our previous studies, we screened the Gram-negative strain of *C. freundii* YNLX and identified its strong ability to degrade HA [20]. Here, we report that we cloned and expressed a novel PL8 hyaluronidase from *C. freundii* YNLX, designated HynACF8, in *E. coli*. We investigated the enzymatic properties of rHynACF8 and the function of its cleavage product.

## 2. Materials and Methods

### 2.1. Bacterial Strain, Vectors, and Reagents

We isolated *C. freundii* YNLX from fish-pond sludge in Sipsongpanna and grew it at 37 °C in Luria–Bertani (LB) medium. We completed strain identification in a previous study [20]. We deposited the strain in the Strains Collection of the Yunnan Institute of Microbiology under registration number YMF3.01173.

We used *E. coli* BL21(DE3) and *p*EASY-E2 vectors, purchased from TransGen (Beijing, China), for gene expression. We used Ni^2+^-NTA agarose, purchased from Qiagen (Valencia, CA, USA), to purify the His_6_-tagged protein. We purchased Genomic DNA isolation kits from Tiangen (Beijing, China) and ClonExpress^®^ II one-step cloning kits from Novizan (Nanjing, China).

We purchased the following reagents: primary substrates hyaluronic acid and chondroitin sulfate (Yuanye, Shanghai, China); a 1,1-diphenyl-2-picrylhydrazyl (DPPH) radical scavenging activity test kit (Congyi, Shanghai, China); a superoxide anion radical (O_2_$\bar{\cdot }$) and hydroxyl radical (·OH) scavenging ability test kit (Solibab, Beijing, China); and a total antioxidant capacity (T-AOC) assay kit with a rapid 2,2′-azinobis(3-ethylbenzthiazoline)-6-sulfonic acid (ABTS) method (Jiancheng, Nanjing, China). All other reagents were of analytical grade (China).

### 2.2. Gene Mining

We sequenced a draft genome of YNLX with the Nanopore method of the, Nextomics Biosciences Co., Ltd (Wuhan, China). We annotated the gene functions based on content from six databases: the Nr, KEGG, GO, KOG/COG, Pfam, and TIGRFAMs databases. We mined a gene designated *hynACF8*, which encodes a putative hyaluronic acid lyase (HynACF8), from the results of KEGG functional annotation.

### 2.3. Sequence Analyses

We performed local sequence analyses using Vector NTI 7.1 (Invitrogen, Waltham, MA, USA). We used online sequence tools BLASTP [21], SignalP [22], and ESPript [23] to search for similar sequences, signal peptides, and multiple sequence alignment mapping, respectively. We performed multiple sequence alignments using the website, MAFFT version 7 (https://mafft.cbrc.jp/alignment/server/, accessed on 1 June 2022) [24]. We performed phylogenetic tree construction (neighbor-joining algorithm and 5.00 bootstraps) with MEGA 7.0.18 software (The Biodesign Institute, Arizona State University, Tempe, AZ, USA) [25].

### 2.4. Heterologous Expression, Purifying, and Identifying Recombinant Hyaluronic Acid Lyase in E. coli

We amplified the HynACF8-encoding gene (*hynACF8*) without the signal peptide-encoding sequence by PCR, using a primer set (5′-TAAGAAGGAGATATACATATGCAGATCGCTACCGAAAATGTAAAT-3′ and 5′-GTGGTGGTGGTGGTGCTCGAGTTTATTTTTAGATAATTCAAAAGAATAACTACTG-3′), which we ligated to *p*EASY-E2, according to the manufacturer’s instructions. The conditions for the induction of recombinant HynACF8 expression and purifying were the same for those used for the β-xylosidase from *Sphingomonas* sp. JB13 in *E. coli* BL21 (DE3) [26]. Sodium dodecyl sulfate-polyacrylamide gel electrophoresis (SDS-PAGE) was used to evaluate the purity of the fractions eluted by Ni^2+^-NTA agarose gel columns. The concentration of SDS-PAGE was 5% polyacrylamide stacking gels and 12% resolving gels.

### 2.5. Enzyme Assay and Substrate Specificity

We employed the ultraviolet (UV) spectrophotometry method to determine the purified recombinant HynACF8 activity, using HA as the substrate. We performed the standard enzyme assay as follows: First, we added 50 μL of rHynACF8 (~0.01 mg/mL) to 450 μL of 0.5% (*w*/*v*) HA substrate in McIlvaine buffer (pH 5.5); we carried out the enzymatic reaction at 35 °C for 10 min and quenched by mixing 3.5 mL of 0.2 M HCl. We determined the activity of the enzyme by spectrophotometrically detecting the formed double bond at 232 nm. Unless otherwise noted, we defined one unit (U) of hyaluronic acid lyase activity as the amount of enzyme needed to form 1 μmol of 4,5-unsaturated uronic acid per minute under the above assay conditions.

(1) $$U=\frac{V_{t}\times \Delta OD_{232\mathrm{nm}}\times N}{d\times \varepsilon \times V_{e}\times T}$$

where V_t_ is the final volume of the reaction mixture, Δ*OD*_232nm_ is the UV absorption at 232 nm, N is the dilution ratio of the enzyme solution, d is 1 cm signifying the thickness of the quartz cuvette, ε is the molar extinction coefficient of unsaturated HA (5500/M/cm) [27], V_e_ is the enzymatic volume added to the reaction mixture, and T is the reaction time.

We studied the substrate specificity of the purified enzyme using different substrates: HA, chondroitin sulfate (CS)-A, dermatan sulfate (DS), polygalacturonic acid, chitin, and peptidoglycan.

### 2.6. Biochemical Characterization of Recombinant Enzyme

We assessed the influence of pH on purified rHynACF8 activity toward HA at 37 °C in pH 3.0–8.0 (McIlvaine buffer) or pH 9.0–10.0 (0.1 M glycine–NaOH). We determined the influence of temperature on purified rHynACF8 activity toward HA at pH 5.5 and 0–60 °C.

We investigated the pH stability of the purified rHynACF8 by detecting the residual activity (pH 5.5, 35 °C) after the incubation of rHynACF8 at pH 3.0–11.0 and 37 °C for 1 h without substrate. We carried out a thermostability assay of rHynACF8 by detecting the residual activity (pH 5.5, 35 °C) after the incubation of the enzyme at pH 5.5 at 40, 50, and 60 °C for various times without HA (pH 5.5).

We evaluated the individual effects on rHynACF8 from adding various metal ions at a final concentration of 1.0 mM in the reaction mixtures. Moreover, we detected the influences of common anions on enzymes in the presence of 10 mM. We measured the activity of rHynACF8 with the addition of salts in McIlvaine buffer (pH 5.5) at 35 °C. We investigated the stability of rHynACF8 by detecting the residual activity at the optimal temperature and pH after incubation of rHynACF8 with the addition of the above salts at pH 5.5 and 35 °C for 60 min.

We performed kinetic experiments on purified rHynACF8 at 35 °C using 0.5–5.0 mg/mL HA as the substrate, prepared in McIlvaine buffer (pH 5.5). We fed the measured data into GraphPad Prism (GraphPad Software, San Diego, CA, USA) and used the results for a nonlinear Michaelis–Menten regression analysis.

### 2.7. Analysis of the Cleavage Products

The cleavage products were prepared by the reaction, including 5 μL of ~0.1 U/mg rHynACF8 and 495 μL of 1% (*w*/*v*) HA at 4 h and 8 h in pH 5.5 and 35 °C. We analyzed the cleavage products by thin-layer chromatography (TLC) and electrospray ionization mass spectrometry (ESI-MS) as previously described [28]. We measured the UV–vis absorption spectra of cleaved products, which ranged in spectrum from 200 to 400 nm, using native HA as a control. We set the UV–vis recording spectrophotometer to zero by McIlvaine buffer (pH 5.5).

### 2.8. Antioxidant Properties of Cleavage Products In Vitro

We conducted an antioxidant test on the cleavage products, i.e., determining the low-molecular-weight HA (LMWHA), based on the reaction and including 1 mL of ~2.6 U/mg rHynACF8 and 5 mL of 5% (*w*/*v*) HA for 5 h in pH 5.5 and 35 °C. We ultra-filtered the reaction mixture in a 10KD ultrafiltration device at a rate of 5000× *g* for 10 min. We collected filtrates to obtain the targeted low-molecular-weight products.

We determined the antioxidant capacity in vitro by the four methods set out in Section 2.8.1, Section 2.8.2, Section 2.8.3 and Section 2.8.4, using products prepared as indicated above.

#### 2.8.1. Assay of ABTS Radical Scavenging Activity

We assessed the ABTS radical scavenging effects of native HA and LMWHA using a total antioxidant capacity (T-AOC) assay kit, according to the manufacturer’s instructions. We measured the absorbance at 405 nm. We used 6-hydroxy-2,5,7,8-tetramethychroman-2-carboxylic acid (Trolox) as our antioxidant standard. We drew the standard curve with 0–1.0 mmol/L Trolox as the ordinate and OD_405nm_ as the abscissa. The assay results are expressed as Trolox-equivalent (TE, mmol/L) via a standard curve. ABTS radical scavenging activity (%) = (TE_sample_/1mmol/L Trolox) × 100.

#### 2.8.2. Assay of DPPH Radicals Scavenging Activity

We evaluated the DPPH radical scavenging effects of native HA and LMWHA using the DPPH radical scavenging activity test kit, according to the manufacturer’s instructions. We measured the absorbance at 517 nm. We prepared the control sample using the same procedure as we used for the test sample, except that we used an equal volume of McIlvaine buffer (pH 5.5). We determined the percentage of DPPH radical scavenging activity by using the following formula: DPPH radical scavenging activity (%) = [(*A_control_*  − *A_sample_*)/*A_control_*] × 100.

#### 2.8.3. Assay of Superoxide Anion Scavenging Activity

We estimated the O_2_$\bar{\cdot }$ radical scavenging effects of native HA and LMWHA using a superoxide anion radical scavenging ability test kit, following the specific steps in the manufacturer’s instruction manual. We measured the absorbance at 530 nm. We prepared the control sample using the same procedure that we used for the test sample, except that we used an equal volume of McIlvaine buffer (pH 5.5). We determined the percentage of O_2_$\bar{\cdot }$ radical scavenging activity by using the following formula: O_2_$\bar{\cdot }$ radical scavenging activity (%) = [(*A_control_ * − *A_sample_*)/*A_control_*] × 100.

#### 2.8.4. Assay of Hydroxyl Radical Scavenging Activity

We estimated the ·OH radical scavenging effects of native HA and LMWHA using a hydroxyl radical scavenging ability test kit, following the specific steps in the manufacturer’s instruction manuals. We measured the absorbance at 536 nm. We prepared the control sample using the same procedure we used for the sample group, except that we used an equal volume of McIlvaine buffer (pH 5.5). In addition, we prepared the blank sample using the same procedure that we used for the control sample, except that we used an equal volume of deionized water instead of an H_2_O_2_ solution. We determined the percentage of ·OH radicals scavenging activity by using the following formula: ·OH radical scavenging activity (%) = [(*A_sample_* − *A_control_*)/(*A_blank_* − *A_control_*)] × 100.

### 2.9. Accession Number

The GenBank accession number of *C. freundii* YNLX *hynACF8* is OM638600.

### 2.10. Statistical Analysis

We express all experimental data as the mean value ± standard deviations in triplicate. We analyzed all variance in the data (one-way ANOVA) using the IBM SPSS statistical 22.0 software (IBM, Chicago, IL, USA). We defined *p* values < 0.05 as statistically significant.

## 3. Results and Discussion

### 3.1. Genome Sequencing and Sequence Analyses

In our previous study, we screened and identified the strain of *C. freundii* YNLX and found that it has a strong ability to degrade HA [20]. We performed the genome sequencing of *C. freundii* YNLX, and the draft genomic sequence was ~5.0 Mbp after data assembly. According to the *KEGG* annotation, we predicted a 2400 bp gene for encoding putative hyaluronic acid lyase. The gene has a GC content of 46.0%, with putative start codon ATG and stop codon TGA. It encodes a 799-residue polypeptide (HynACF8). The protein sequence of the deduced HynACF8 has a signal peptide ranging from M1 to A20 and a catalytic domain that belongs to the PL8 hyaluronic acid lyases.

The results of BLASTP analysis from the NCBI database revealed that HynACF8 had the highest identity, 99.87%, with the hypothetical hyaluronic acid lyase from *Yersinia enterocolitica* (CFB71160), and less than 50% identities with the experimentally characterized PL8 hyaluronic acid lyases. Among those lyases, HynACF8 had the highest similarity with PL8 HCLase from *Vibrio* sp. FC509 (AIL54323), with an identity of 48.9% [19].

**Figure 1:**
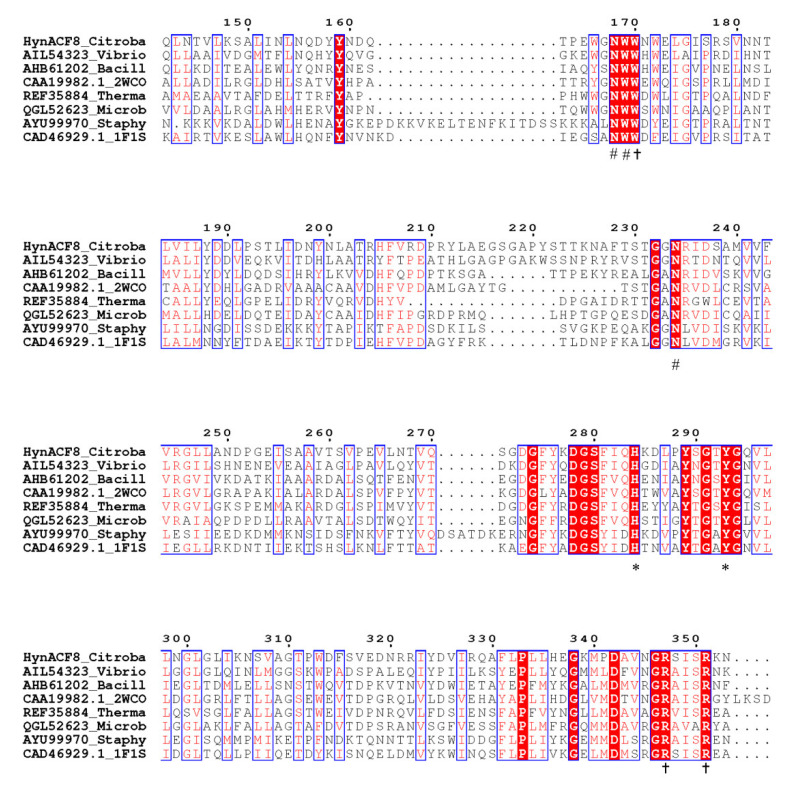
Partial multiple-sequence alignment of HynACF8 with PL 8 hyaluronic acid lyase. Sequences are as follows (including accession numbers): HCLase from *Vibrio* sp. FC509 (AIL54323), HAase-B from *Bacillus* sp. A50 (AHB61202), PL8Hyal from *Streptomyces coelicolor* A3(2) (CAA19982, 2WCO), TcHly8C from *Thermasporomyces composti* DSM22891 (REF35884), HCLaseM from *Microbacterium* sp. H14 (QGL52623), HylSA from *Staphylococcus aureus* (AYU99970), and HylB from *Streptococcus agalactiae* NEM316 (CAD46929, 1F1S). Identical and similar amino acids are shaded in boxes, respectively. Catalytic amino acid residues are marked with asterisks (*); the −1 substrate-binding subsite residues are marked with a cross (†); the +1 substrate-binding subsites are marked with a pound sign (#).

The alignment of HynACF8 and the experimentally characterized hyaluronic acid lyases revealed the conserved catalytic residues (Figure 1). The conserved catalytic residues of HynACF8 are H284 and Y293 as the Brønsted base and acid, respectively. The −1 substrate-binding subsites of HynACF8 are R347 and R351. The +1 substrate-binding subsites of HynACF8 are N168, W169, and N234 [18,29]. The connected sequences of nearby functional sites have a high degree of diversity, which may be an important factor interfering with the biochemical properties of the enzyme.

### 3.2. Expression and Purification of rHynACF8

We successfully expressed the *hynACF8* gene, without the signal peptide sequence, in *E. coli* BL21 (DE3). We purified the His-tagged rHynACF8 to electrophoretic homogeneity by Ni^2+^–NTA affinity chromatography. A single band of approximately 85 kDa migrated in SDS-PAGE (Figure S1), a result that confirmed the calculated molecular weight of HynACF8 without the signal peptide sequence.

### 3.3. Substrate Specificity

We studied the substrate specificity of rHynACF8 according to the composition of monosaccharides of HA using different substrates formed by N-acetyl-glucosamine (GlcNAc) or uronic acid (UA) [3]. Our results showed that purified rHynACF8 could degrade HA and CS-A with 100.0% and 31.9% relative activities, respectively (Figure S2). However, it was not active toward DS, polygalacturonic acid, chitin, or peptidoglycan. Polysaccharide lyases act on polysaccharides containing a hexose oxidized at C-5 position to a carboxylic group and cleave the glycosidic bond at the C-4 position using β-elimination mechanism [30]. Most of the PL8 lyases are able to degrade glycosaminoglycans, such as HA, CS, and DS. In general, hyaluronic acid lyase can be classified in the same subfamily because of the stronger ability to degrade HA than CS [14,16,31]. Therefore, rHynACF8 is a typical hyaluronic acid lyase based on functional analysis.

### 3.4. Biochemical Characterization

**Figure 2:**
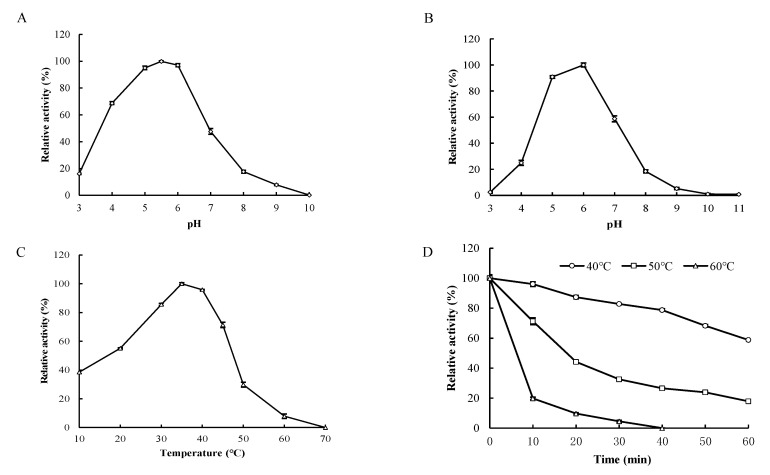
Enzymatic properties of purified rHynACF8. (**A**) pH-dependent activity. (**B**) pH-dependent stability. (**C**) Temperature-dependent activity. (**D**) Temperature-dependent stability. Error bars represent the means ± SD (*n* = 3).

Purified rHynACF8 has an apparent optimum pH of 5.5 at 37 °C (Figure 2A) and is stable at pH ranging from 5.0 to 6.0 for 1 h (Figure 2B). When assayed at pH 5.5, it showed apparent optimal activity at 35 °C, and retained ~30% and 50% of its maximum activity at 10 and 20 °C (Figure 2C). Purified rHynACF8 is stable below 40 °C, and its half-life is ~15 min at 50 °C (Figure 2D).

We investigated the effects of various metal ions on the activity of rHynACF8 at a final concentration of 1 mM (Table 1). We always added metal ions to the reaction in the form of metal salts. To eliminate the influence of anions in metal salts on enzyme activity, we determined the effect of 10 mM NaCl, Na_2_SO_4_, and NaAc on enzymatic activity and stability. The results indicated that they rarely had no effect on enzymatic activity and stability (Table S1). It followed that metal ions in salts played a major role in the catalytic activity of rHynACF8 in the reaction mixture. As shown in Table 1, rHynACF8 was completely inhibited by Fe^3+^ and partially inhibited by Al^3+^. However, the catalytic activity of rHynACF8 was not substantially affected by the presence of other metal ions and EDTA (more than 75.5% activity), especially for the common Zn^2+^.

We measured the specific activity and kinetic parameters of purified rHynACF8 at pH 5.5 and 35 °C using the UV spectrophotometry method. Its specific activity toward 2 mg/mL HA is 12.8 ± 0.1 U/mg, and the *K_m_*, *V_max_*, and *k_cat_* of the enzyme are 1.5 ± 0.01 mg/mL, 20.1 ± 0.2 U/mg, and 30.9 ± 0.5 /s, respectively (Figure S3). Before comparing the catalytic activity of hyaluronic acid lyase, we should consider the enzyme assay method. In previous studies, turbidimetry and UV spectrophotometry were the methods commonly used to measure hyaluronic acid lyase [11]. The amount of residual substrate was determined by turbidimetry, whereas the amount of double bond in the generated product was determined by UV spectrophotometry. As the molecular weights of the polymer and hyaluronate oligosaccharides are variable, the data determined by the two methods cannot be unified through conversion. Therefore, we could not compare the results with each other.

**Figure 3:**
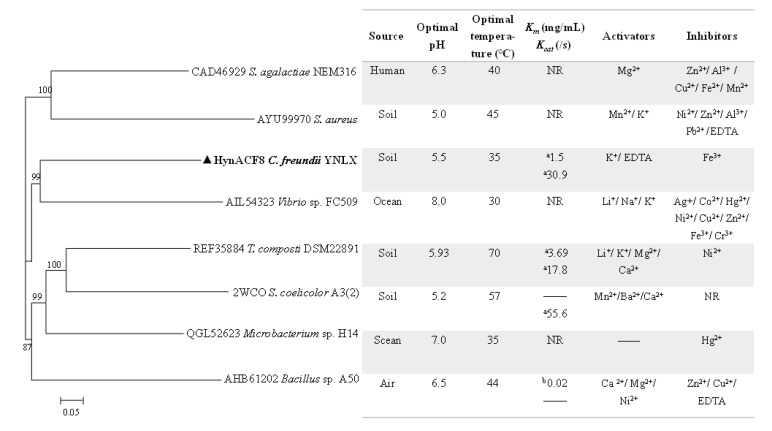
Phylogenetic tree constructed using the amino acid sequences of experimentally characterized hyaluronic acid lyase. ^a^ UV spectrophotometry. One unit (U) of hyaluronic acid lyase activity was defined as the amount of enzyme formed by 1 μmol unsaturated double bonds per minute using HA as the substrate under certain conditions. ^b^ Bovine albumin turbidimetry. One unit (U) of enzymatic activity was defined as the amount of enzyme equal to that of 1 U standard hyaluronidase for splitting hyaluronic acid in 30 min under specific conditions.

A comparison of the properties of rHynACF8 and the experimentally characterized PL8 family recombinant hyaluronic acid lyases is shown in Figure 3. With regard to pH, the environment of microbial origin, rather than the phylogenetic relationships, played a key role [32]. The optimum pH of soil-derived hyaluronic acid lyases is less than 6.0, whereas the optimum pH levels of sea-derived hyaluronic acid lyases are all more than 7.0. The results of phylogenetic tree analysis showed that enzymes with a similar optimal pH do not belong to the same evolutionary branch, suggesting that they might adopt different molecular strategies to adapt to similar environments (Figure 3). Regarding the effects of various metal ions on enzymatic activity, HynACF8, like HCLaseM from *Microbacterium* sp. H14 and TcHly8C from *T. composti* DSM22891, is less affected by metal ions than that of HCLase from *Vibrio* sp. FC509 and HylS from *S. aureus*. Moreover, most of the enzyme activity of hyaluronic acid lyases are susceptible to divalent metal ions, such as common Zn^2+^, while the enzyme activity of HynACF8 is almost unaffected by divalent metal ions. The special metal ions resistance makes HynACF8 a candidate for further basic research and application. Furthermore, hyaluronidases from microorganisms will be a hot area of research because of their diverse enzymatic properties and different relationship between structure and function.

### 3.5. Degradation Pattern and Cleavage Products of rHynACF8

**Figure 4:**
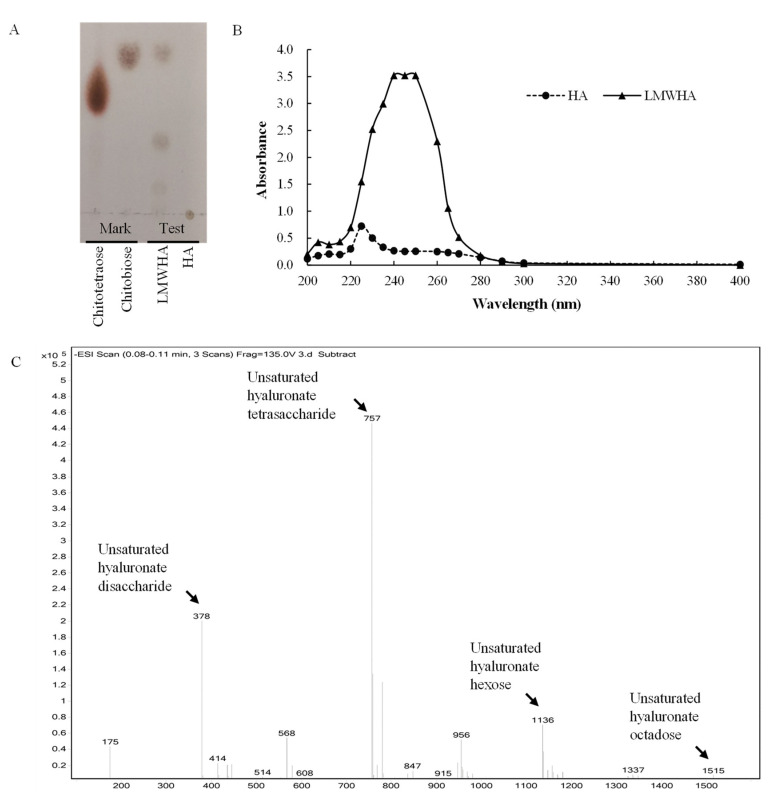
Analysis of the cleavage products of rHynACF8. (**A**) TLC; (**B**) UV–vis absorption spectra; (**C**) ESI-MS.

To determine the degradation pattern, we analyzed the cleavage products of HA produced by rHynACF8 using TLC, ESI-MS, and UV–vis absorption spectra. According to the TLC results, rHynACF8 degrades the substrate HA into LMWHA (Figure 4A). The degradation pattern of rHynACF8 is similar to that of the endolytic mode. The endo-type enzymes produce higher-diversity molecular mass oligosaccharides and smaller oligomers, such as HCLase from *Vibrio* sp. FC509 [19] and HCLaseM from *Microbacterium* sp. H14 [14]. By further determining the composition of LMWHA produced by rHynACF8, the negative ESI-MS spectrum showed dehydrogenated molecular ion peaks at *m*/*z* 378, 757, 1136, and 1515 (Figure 4C). The *m*/*z* value corresponds to the mass of the unsaturated hyaluronan di-, tetra-, hexa- and octa-saccharide residue, minus the mass of a hydrogen ion. The UV–vis absorption spectra indicated that LMWHA has a strong absorption peak at 230–260 nm compared with that of the substrate (Figure 4B). This result confirmed the view that PL8 hyaluronic acid lyases can degrade HA to unsaturated disaccharide units or their repeating unit oligosaccharides [29]. In summary, HynACF8 is an endo-acting hyaluronic acid lyase that produces unsaturated di-, tetra-, hexa- and octa-saccharides—mainly unsaturated hyaluronic acid disaccharides and tetrasaccharides.

### 3.6. Antioxidant Properties of Cleavage Products

**Figure 5:**
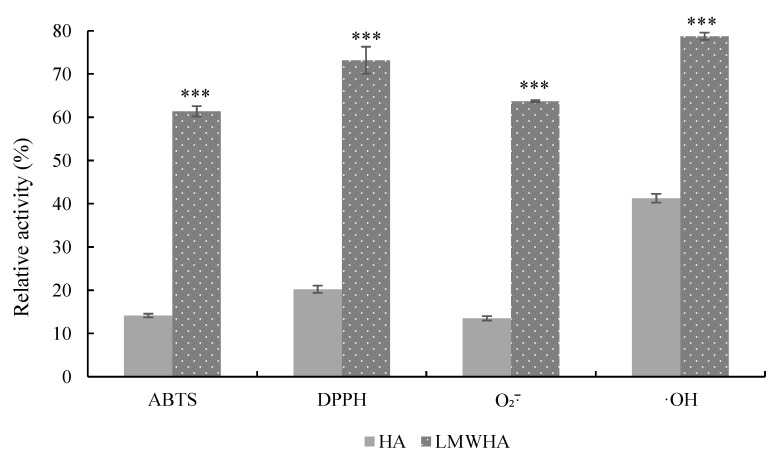
Antioxidant activity of the cleavage products of rHynACF8. The scavenging ability of LMWHA and HA for each free radical is individually labeled according to the significant difference test; “***” refer to an activity with the significant difference test (*p* < 0.001).

The ABTS, DPPH, O_2_$\bar{\cdot },$ and ·OH radical scavenging activities are the most common indicators for analyzing the antioxidant properties of carbohydrates in vitro [33]. As shown in Figure 5, the inhibition rates of ABTS, DPPH, O_2_$\bar{\cdot },$ and ·OH radicals were 61.40%, 73.21%, 63.70%, and 78.74% by LMWHA, respectively. In addition, the inhibition rates of the above radicals by HA were only 14.14%, 20.21%, 13.50%, and 41.27%, respectively. We observed a significant difference (*p* < 0.001) between LMWHA and HA in antioxidation ability, which showed that the radical scavenging capacity of LMWHA is significantly stronger than that of HA per the results of the above four indicators. The antioxidant ability of LMWHA is better than that of HA [34,35,36]. However, the enhanced antioxidant capacity of the cleavage products of hyaluronic acid lyases was not clearly explained in terms of molecular properties.

**Figure 6:**
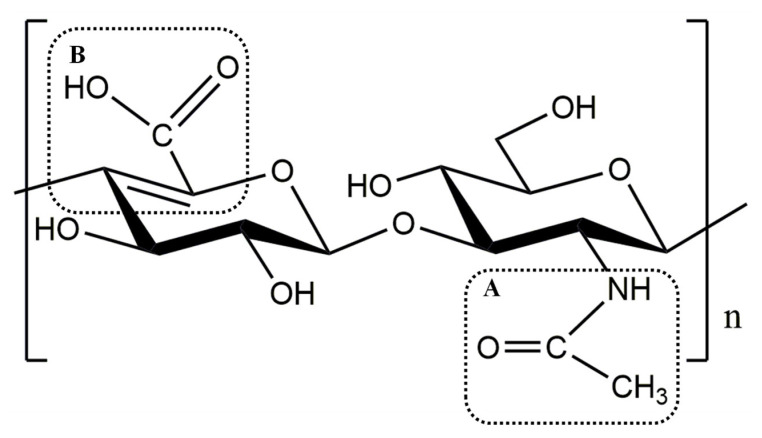
General structure of unsaturated hyaluronic acid oligosaccharide. GlcUA, D-glucuronic acid. GlcNAc, N-acetyl-D-glucosamine. (**A**) Acetylamino groups of GlcNAc; (**B**) conjugated double bond between Δ4,5-unsaturated bond and C_6_ carboxyl groups of GlcUA.

The radical scavenging ability of chemical compounds depends on their structures. The compounds containing resonating structures allow the odd electron to be delocalized over the whole molecule, thus maintaining stability even in free-radical form [37]. The resonating structures have free carboxyl groups, carbonyl groups, amino groups, conjugated double bonds, etc. [38]. The two main reasons for LMWHA being a strong antioxidant are indicated by its structure (Figure 6). One reason was that as the molecular weight of HA decreases, the intramolecular hydrogen bonds among carboxyl groups of GlcUA and acetylamino groups of GlcNAc transform into intermolecular hydrogen bonding [3]. As a result, functional groups of HA with radical scavenging ability are released. Another possible reason is related to a new double bond formed between C_4_ and C_5_ of GlcUA of HA during cracking. Then, an increasing conjugated and delocalization effect forms between the Δ4,5-unsaturated bond and C_6_ carboxyl groups of GlcUA, which improves the radical scavenging ability of the cleavage product. In summary, LMWHA cleaved by the hyaluronic acid lyase has a more beneficial molecular structure for antioxidation than HA.

## 4. Conclusions

In this study, the PL8 hyaluronic acid lyase, HynACF8, was isolated from a Gram-negative *Citrobacter* strain originating from fish-pond sludge, which we expressed in *E. coli*. rHynACF8 has metal –ion resistance, especially for divalent metal ions. rHynACF8 is an endo-acting hyaluronic acid lyase and produces unsaturated oligosaccharides, especially in unsaturated hyaluronic acid disaccharides and tetrasaccharides. Unsaturated oligosaccharides produced by HynACF8 have a stronger antioxidation ability than uncleaved HA because of the structures of free carboxyl groups, amino groups, and conjugated double bonds. Therefore, HynACF8 is a novel powerful enzyme for further basic research and potential applications in several areas, including health food, cosmetics, and medical therapy.

## Figures and Tables

**Table 1:** Effects of various metal ions and chemical reagents on purified rHynACF8.

Substance	Relative Activity (%) ^a^	Substance	Relative Activity (%) ^a^
none	100.0 ± 0.8	MgSO_4_	99.4 ± 2.2
KCl	119.9 ± 1.4	MnSO_4_	98.8 ± 0.6
LiCl	109.4 ± 1.0	ZnSO_4_	97.5 ± 0.9
NaCl	109.3 ± 1.1	NiSO_4_	93.4 ± 0.5
CaCl_2_	106.9 ± 0.6	FeSO_4_	79.1 ± 0.4
CoCl_2_	96.4 ± 0.7	CuSO_4_	75.5 ± 1.3
AlCl_3_	55.5 ± 1.1	PbAc	77.1 ± 1.9
FeCl_3_	0	EDTA	116.3 ± 1.9

^a^ Values represent the means ± SD (*n* = 3) relative to the untreated control sample.

## Data Availability

The data in this study are available within manuscript and Supplementary Materials.

## Supplementary Materials

The following supporting information can be downloaded at: https://www.mdpi.com/article/10.3390/foods11131989/s1, Figure S1: SDS-PAGE analysis; Figure S2: The illustration of substrate structures degraded by purified rHynACF8; Figure S3: Kinetic characterization of purified rHynACF8; Table S1: Effects of anions in metal salts on purified rHynACF8.
