# Supplementary material for: Biochemical and Molecular Characteristics of a Novel Hyaluronic Acid Lyase from Citrobacter freundii
Source: Foods. 2022 Jul 5;11(13):1989. doi: 10.3390/foods11131989 (PMC9265501; doi:10.3390/foods11131989)
Supplement: Supplementary file 1 [file foods-11-01989-s001.zip › foods-1736711-supplementary.pdf]

Supporting information for publication

Biochemical and molecular characteristics of a novel hyaluronate lyase from *Citrobacter freundii*

Xinyue Li[2,], Fang Li[2,] , Junhao Ma[2], Mingjun Li[2], Xi Lei[2], Xianghua Tang[1, 2, 3, 4], Qian Wu[1, 2, 3, 4], Rui Zhang[1, 2, 3, 4, *], Zunxi Huang[1, 2, 3, 4, *]

[1]Engineering Research Center of Sustainable Development and Utilization of Biomass Energy, Ministry of Education, Yunnan Normal University, Kunming, China;

[2]College of Life Sciences; Yunnan Normal University; Kunming, China;

[3]Key Laboratory of Yunnan for Biomass Energy and Biotechnology of Environment, Yunnan Normal University, Kunming, China;

[4]Key Laboratory of Yunnan Provincial Education Department for Plateau Characteristic Food Enzymes, Yunnan Normal University, Kunming, China

Running title: A novel hyaluronate lyase from *Citrobacter freundii*

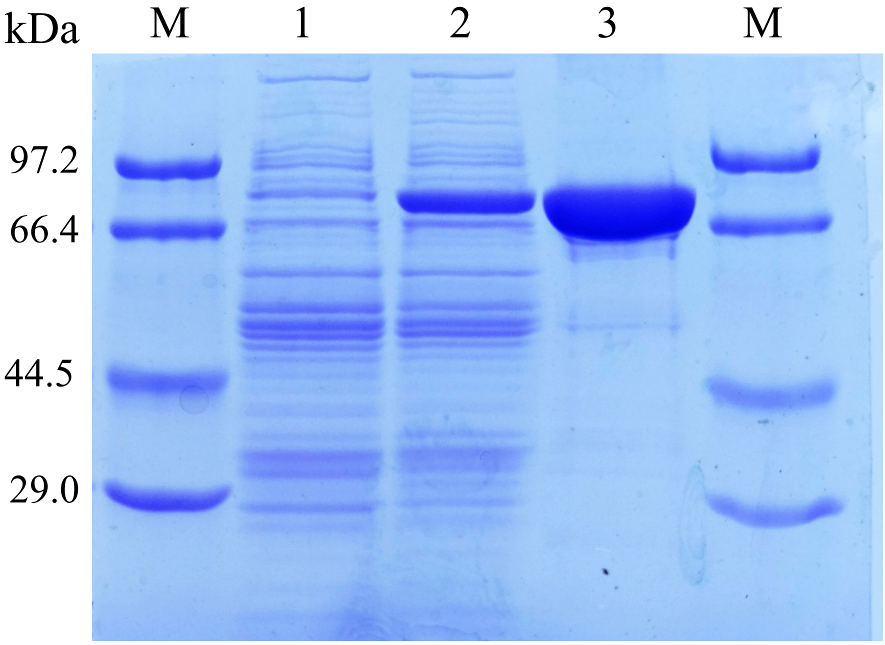

Figure S1. SDS-PAGE analysis.

Lanes: M, protein molecular weight marker; 1 culture supernatant of induced transformant harboring the empty plasmid, 2 culture supernatant of the induced transformant harboring *p*EASY-*hynACF8*, 3 purified recombinant HynACF8 by Ni^{2+}–NTA.

A

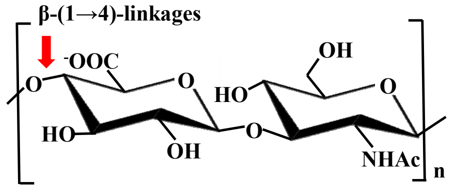

Hyaluronic acid

B

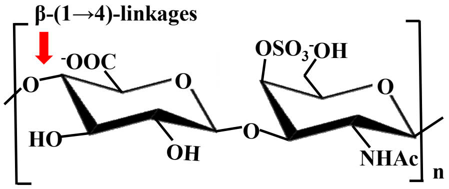

Chondroitin sulfate A

Figure S2. The illustration of substrate structures degraded by purified rHynACF8.

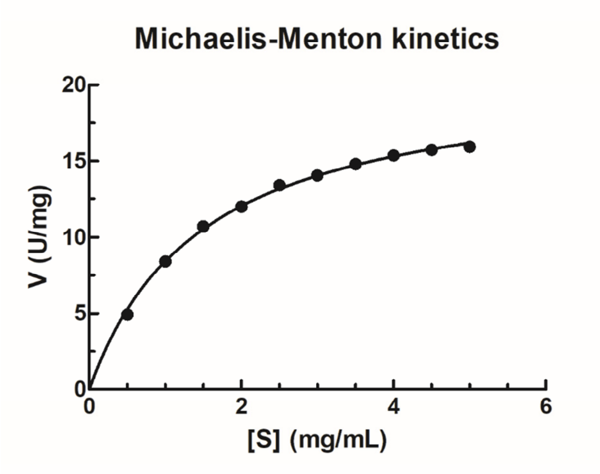

Michaelis-Menten kinetics	Table of results
Best-fit values	
Vmax	21.05
Km	1.498
Std. Error	
Vmax	0.2192
Km	0.04445
95% Confidence Intervals	
Vmax	20.60 to 21.50
Km	1.407 to 1.589
Goodness of Fit	
Degrees of Freedom	28
R2	0.9958
Absolute Sum of Squares	1.489
Sy.x	0.2306
Constraints	
Km	Km > 0.0
Number of points	
Analyzed	30

Figure S3. Kinetic characterization of purified rHynACF8.

Table S1. Effects of anions in metal salts on purified rHynACF8

substance	activity-relative activity (%)[a]	stability-relative activity (%)[a]
NaCl	126.9±2.9	79.4±1.6
Na_2SO_4	84.2±5.0	140.7±4.7
NaAc	88.6±4.3	102.5±3.4

[a] Values represent the means ± SD (n = 3) relative to the untreated control sample.
